# Atmospheric Pressure and Onset of Episodes of Menière’s Disease - A Repeated Measures Study

**DOI:** 10.1371/journal.pone.0152714

**Published:** 2016-04-20

**Authors:** Robert Gürkov, Ralf Strobl, Nina Heinlin, Eike Krause, Bernhard Olzowy, Christina Koppe, Eva Grill

**Affiliations:** 1 Department of Otorhinolaryngology Head and Neck Surgery, Ludwig-Maximilians-Universität München, Marchioninistr. 15, 81377 Munich, Germany; 2 German Center for Vertigo and Balance Disorders, Ludwig-Maximilians-Universität München, Marchioninistr. 15, 81377 Munich, Germany; 3 Institute for Medical Information Processing, Biometrics and Epidemiology (IBE), Ludwig-Maximilians-Universität-München, Marchioninistr. 17, 81377 Munich, Germany; 4 German Meteorological Service, Frankfurter Str. 135, 63067 Offenbach, Germany; University of Regensburg, GERMANY

## Abstract

**Background:**

External changes of air pressure are transmitted to the middle and inner ear and may be used therapeutically in Menière’s disease, one of the most common vertigo disorders. We analyzed the possible relationship of atmospheric pressure and other meteorological parameters with the onset of MD vertigo episodes in order to determine whether atmospheric pressure changes play a role in the occurrence of MD episodes.

**Methods:**

Patients of a tertiary outpatient dizziness clinic diagnosed with MD were asked to keep a daily vertigo diary to document MD episodes (2004–2009). Local air pressure, absolute temperature and dew point temperature were acquired on an hourly basis. Change in meteorological parameters was conceptualized as the maximum difference in a 24 hour time frame preceding each day. Effects were estimated using additive mixed models with a random participant effect. We included lagged air parameters, age, sex, weekday and season in the model.

**Results:**

A total of 56 persons (59% female) with mean age 54 years were included. Mean follow-up time was 267 days. Persons experienced on average 10.3 episodes during the observation period (median 8). Age and change in air pressure were significantly associated with vertigo onset risk (Odds Ratio = 0.979 and 1.010). We could not show an effect of sex, weekday, season, air temperature, and dew point temperature.

**Conclusions:**

Change in air pressure was significantly associated with onset of MD episodes, suggesting a potential triggering mechanism in the inner ear. MD patients may possibly use air pressure changes as an early warning system for vertigo attacks in the future.

## Introduction

Menière’s Disease (MD) is defined as the idiopathic syndrome of endolymphatic hydrops, characterized by sudden spells of rotatory vertigo and hearing loss, tinnitus and aural pressure, caused by a distension of the endolymphatic space of the inner ear. With a prevalence of 200 to 500 per 100.000 [1, 2], it is a rather common inner ear disorder and causes a considerable socioeconomic burden due to its chronic and incurable nature.

Typically, MD attacks occur in episodes and are sometimes preceded by aural symptoms. The onset of MD attacks in most patients, however, is unpredictable, even though various possible triggering factors have been reported, e.g. mental stress, weather changes, atmospheric pressure changes during ropeway or car drives in alpine environments. Acute MD is one of the most debilitating known nonlethal diseases [3]. Vertigo has the major impact on the quality of life in MD patients [4], and the psychological effect of unexpected paroxysms of vertigo is even more distressing to the patients than the vertigo itself [5].

To date, the precise pathophysiologic mechanism of an MD attack is unresolved. An elevated hydrostatic pressure associated with the endolymphatic hydrops (distension of the membraneous labyrinth containing the sensory organs of the inner ear) is discussed as the primary pathophysiological event. Schuknecht suggested the membrane-rupture-theory to explain acute attacks in Menière’s disease [6]. Some features of nystagmus during acute attacks are more consistent with Tonndorf’s theory of raised endolymphatic pressure [7, 8]. It is known that vestibular receptors are pressure-sensitive [9]. It is likely that a valve-like structure discovered by Bast in 1928 plays a role in endolymph pressure regulation [10], and its dysfunction has been proposed to cause endolymphatic hydrops [11]. A perilymphatic pressure increase (caused e.g. by an atmospheric pressure increase) is transmitted to the endolymph space across the very thin and compliant boundary membranes of the endolymphatic space, and a physiological opening of Bast’s valve may allow endolymphatic outflow in order to protect the vestibular sensory organs from excessive pressure. Data from experimental perilymphatic injections also suggest the presence of a one-way valve between the labyrinth and the endolymphatic sac [12]. The precise mechanism, however, of the regulation of the inner ear fluid volume and electrolyte composition, has not yet been elucidated satisfactorily. Another possible way of balancing volume or pressure changes brought about by internal or external disturbances is suspected to be exerted by a local paracrine mechanism involving the atrial natriuretic peptide (ANP) system, since both ANP and its receptors have been shown to be present and synthesized within the inner ear [13, 14].

Recently, a local overpressure therapy has been shown to be effective for the prophylactic treatment of MD [15, 16]. This treatment consists of small pressure oscillations applied to the inner ear fluid system via the middle ear and the round and oval window. Changes in middle ear pressure in the range of tens of millibars are transmitted to the perilymph almost without any loss; Pressure transfer also occurs from the external ear canal via the middle ear to the perilymph, albeit with some loss [17]. Furthermore, it has been shown that even small atmospheric pressure fluctuations in the range of tens of Pascals are also transmitted to the middle ear [18] via the pars flaccida of the tympanic membrane. Changes in middle ear pressure also have an impact on vestibular afferent firing rates [19]. Those findings indicate that external atmospheric pressure changes may be transmitted into the inner ear, eliciting pressure changes in the inner ear which subsequently may have an effect on the onset and frequency of MD attacks.

The objective of this study was therefore to examine whether MD attacks are preceded by atmospheric pressure changes.

## Materials and Methods

### Setting and sample

We conducted a prospective cohort study on a convenience sample of patients referred to a tertiary care vertigo and balance clinic in Munich, Germany, between October 2004 and July 2009. We included individuals aged 18 or above living in the Metropolitan Area of Munich. The residence and workplace had to be within a maximal distance of 50 km from the Munich Meteorological station. In addition, the distance to the closest meteorological station of the German Weather Service was analyzed, and 11 of the 56 patients were living closer to the Augsburg meteorological station, for these cases the meteorological data from the Augsburg station were used. All participants had a diagnosis of definite unilateral Menière’s disease (MD) and were treated with standard-dose oral Betahistine (72 mg daily). Our criteria for unilateral MD were: absence of significant contralateral hearing loss (> 30 dB), absence of contralateral aural syptoms during attacks. We excluded bilateral MD cases because in our experience they usually have a longer disease duration than the “typical” unilateral MD patient, and their vertigo symptoms are less well-defined temporally, i.e. they tend to have more continuous and motion-dependent vertigo symptoms (probably due the bilateral vestibular impairment) and less clearly-defined sudden spells of rotatory vertigo. Previous destructive therapy (e.g. chemical labyrinthectomy) was excluded. The study was reviewed by the data security official of the University Hospital and a waiver for a formal ethical review process was granted (Ref.Nr. 847, Ethics committee of the University of Munich). Participants had given informed written consent. The study was was conducted in accordance with the principles of the Declaration of Helsinki.

Munich is the capital of the federal state of Bavaria and with 1.5 million inhabitants and the third biggest city in Germany. It is situated on the 48th degree of latitude (north) and 11.5 degree longitude (east). Munich extends between 480m and 580m above sea level.

Munich belongs to the transition zone between humid oceanic climate in the west and dry continental climate in the east. The average temperature in Munich in the period 2004–2009 was 9.7°C. The coldest month was January with 0.3°C average temperature. July was the warmest month in Munich, with an average temperature of 19.6°C.

The mean annual precipitation amount was 935 mm between 2004 and 2009. There is no dry season in Munich, however mean monthly precipitation totals varied from 46mm in February to 136mm in July. Between 2004 and 2009 the average annual sunshine duration was 1858 hours.

### Measurements

#### Diagnosis

Diagnosis of definite MD according to the AAO-HNS guidelines [20] was confirmed based on complete neuro-otological work-up carried out by experienced neurotologists. Neurotological examination included a comprehensive battery of bedside tests, audiologic and vestibular function tests, cranial MRI to rule out neoplastic and central pathology and, if necessary, further imaging techniques and consultation of other medical specialities (e.g. neurology, psychiatry or ophthalmology).

#### Outcome

All participants were invited to keep a daily vertigo diary to document occurrence and intensity of MD attacks. Intensity of vertigo was measured on a 5-point scale ranging from zero (no vertigo) to four (severe vertigo). Vertigo-free days were scored as 0. Days with a mild attack were scored as 1. Moderately severe attacks lasting more than 20 minutes were scored as 2; severe attacks lasting an hour or more or accompanied by nausea or vomiting were scored as a 3. A level 4 attack was the worst attack ever experienced to date. For the purpose of this study we defined an attack day as a day with an intensity of two or higher. A MD episode would start with an attack day that was preceded by at least three days without attacks. Thus we concentrated on assessing the risk factors for the onset of an MD episode. The occurrence of drop attacks (Tumarkin attacks) was not specifically recorded. Age and sex of MD patients were retrieved from medical charts and included as covariates.

#### Exposure

Meteorological data were obtained from the Meteorological stations of Munich and Augsburg on an hourly basis for the years 2004–2009. Air pressure was measured in hectopascal (hPa). We assigned to each person the meteorological data of the nearest the Meteorological station.

We conceptualized change in air pressure as the maximal change in air pressure within a 24 hour time frame. Thus, for each hour of the day the difference in air pressure to each of the preceding 24 hours was calculated. The highest absolute difference for each hour was retained. Change in air pressure per day was then defined as the highest absolute difference of all 24 hours of this day (*DiffPressure*_*0*_). This absolute difference was assigned a positive sign, if it corresponded to an increase of air pressure within the last 24 hours, and a negative sign, if it corresponded to a decrease. Additionally, we examined the effect of mean air pressure per day (*MeanPressure*_*0*_). We also examined the effect of lagged differences in air pressure and mean air pressure on the risk of a MD episode, i.e. lags of one day (*DiffPressure*_*1*_ and *MeanPressure*_*1*_), two days (*DiffPressure*_*2*_ and *MeanPressure*_*2*_), or three days (*DiffPressure*_*3*_ and *MeanPressure*_*3*_).

We dichotomized the parameter *DiffPressure*_*k*_ into “Pressure Increase” and “Pressure Decrease” as *DiffPressure*_*k*_ *> 0*.

Thermal discomfort is an important cause of meteoropathological disturbances [21]. To exclude the possibility of a more general biometeorological effect, we also investigated temperature and dew point temperature as potential predictors of MD episodes. Variables indicating changes in temperature and dew point were treated analogously to pressure.

### Data analysis

We report means, minimum, maximum, quartiles and standard deviations or confidence intervals for continuous variables and percentages and frequencies for categorical variables. We visualize the relationship of time, air pressure and risk of MD episode using a local polynomial regression smoother (loess) [22]. Here, the fit of a point *x* is based on each point in a neighbourhood of *x*. Points close to *x* have a higher weight, i.e. a higher impact on the fit. The weights decrease in a tricubic way with increasing distance to *x*, that is proportional to *(1 - (dist/maxdist)^3)^3)*. The size of the neighbourhood can be controlled by a prespecified parameter *k*. For *k = 1* the neighbourhood includes all points, for *k < 1* the proportion *k* of all points is used. By using a loess smoother we are able to capture important patterns in the data, without being distracted by noise in the measurements. In addition, as the loess smoother is based on local linear least squares regressions, we can construct pointwise confidence intervals for each fitted point. Using this method we visualized the association of meteorological parameters and time since study inclusion as exposure and prevalence of onset of an MD episode as outcome variable. Autocorrelation plots were used to evaluate collinearity among the lags of meteorological parameters. They display the cross-correlation of a variable with the lagged (i.e. time-shifted) values of itself. Lagged values with correlations greater than 0.4 were not included simultaneously into the same models because of them being susceptible for multicollinearity, i.e. for variance inflation.

Seasonal effects were evaluated by plotting meteorological parameters over time, also using the loess smoother.

To assess the effects of covariates and air pressure on the risk of onset of an MD episode we used generalized linear mixed models with a logit link function, i.e. the logit-transformed probability is modelled as a linear relationship with the predictor variables. By including a random participant effect we account for differences in the individual risk for an MD episode among all participants.

Potential covariates were time, age, sex, weekday, season and air pressure.

The time variable was modelled as either a constant term, i.e. no change in risk over the observation period, a linear term or using polynomials (up to 3 degrees) to allow for nonlinear exposure—response functions. In addition to including concurrent meteorological parameters in the final models, all models included variables indicating lagged meteorological parameters for up to three days. All meteorological variables were investigated either linearly or using polynomials (up to 3 degrees) to allow for nonlinear exposure—response functions.

Model selection was based on comparison of the Akaike Information Criterion (AIC). AIC = Deviance + 2(number of parameters in the model). Lower AIC indicates better fit. We also report the more conservative Bayesian Information Criterion (BIC). Fixed effects and variance components were tested for statistical significance using the z-statistics. The coefficients are presented as the odds ratios together with p-values. Odds ratios are calculated as the exponential of the coefficients of each of the predictors, e.g. for continuous variables a change by one in the predictor has an effect on the odds ratio by the exponential of the coefficient. An odds ratio higher than one corresponds to an increased risk for the onset of an MD episode given all other covariates are held constant, e.g. with the same time, age, gender, or other meteorological covariates.

To show the results on the individual level, we additionally report statistics on standard diagnostic parameters for each patient, i.e. accuracy, sensitivity (SEN), specificity (SPEC), the positive predictive value (PPV) and the negative predictive value (NPV) of meteorological parameters as a prediction tool for the risk of onset of an MD episode.

In order to investigate the influence of the chosen cut-off, we repeated the analysis and defined an attack day as a day with an intensity of one or higher, three or higher, and four. All sensitivity analyses are presented in tables A-C in S1 Appendix.

All analyses were done using R [23].

## Results

### Study population

During the study period, 128 patients with Menière’s disease visited our clinic at least twice. Of these, 101 patients reported a completed vertigo diary over a minimum of 30 days. From these, those patients were selected for inclusion in the study who are living within a 50-km radius of Munich City, i.e. 57 subjects. One participant was excluded for having chronic vertigo, i.e. a vertigo score > = 2 on every day. All following analysis are based on the remaining 56 individuals. These 56 individuals with a median age of 51 years (59% female) completed 14936 entries in the vertigo diary. We excluded all entries with a missing value in the outcome (n = 934) and all observations within an MD episode (n = 2631). Thus, 11371 valid entries were available for analyses.

Median follow-up time was 240 days. Individuals experienced on average 10.3 MD episodes during the observation period (median 8) and completed on average 203 entries (median 197). The highest fraction of study entries (20%) occurred in October, and the lowest fraction in January and May (4% each). We could not show a significant difference in the distribution of the month of study entries (p = 0.08845). Characteristics of the 56 individuals at time of inclusion are described in Table 1.

**Table 1:** Baseline characteristics of the study population (n = 56).

*Variable*	*mean ± SD*	*min*	*1^St^Quart*	*median*	*3^rd^Quart*	*max*	*95%-CI*
	n (%)						
Age (yrs)	54.3 **±** 15.5	19.5	45.8	51.4	66.6	85.1	(50.1, 58.4)
Gender: Female	33 (59%)	-	-	-	-	-	(45%, 72%)
Length of Follow-Up (d)	266.7 **±** 152.6	32	170.5	239.5	336	806	(225.8, 307.6)
Number of Episodes	10.3 **±** 10.4	0	3	8	14.25	60	(7.5, 13.1)
Stage							
1	13 (23%)	-	-	-	-	-	-
2	9 (16%)						
3	24 (43%)						
4	10 (18%)						

### Atmospheric pressure and MD episodes

Table 2 describes the statistical characteristics of atmospheric pressure parameters for the whole study period (2004–2009). Mean air pressure for both meteorological regions *MeanPressure*_*0*_ was 1017.7 hPa ranging from 984 hPa to 1044 hPa (Table 2). Mean change in air pressure *DiffPressure*_*0*_ ranged from -30.9 hPa to 26.8 hPa with on average -0.20 hPa for Munich and from -31.1 hPa to 27.1 hPa with on average -0.22 hPa for Augsburg. We detected no autocorrelation over 0.4 for *DiffPressure*, but for *MeanPressure* (S1 Fig).

**Table 2:** Atmospheric pressure parameters in hPa for Augsburg and Munich.

Variable	mean ± SD	min	1^St^Quart	median	3^rd^Quart	max
**Munich**						
DiffPressure_0_	-0.20 **±** 9.47	-30.9	-7.1	-3.9	7.2	26.8
MeanPressure_0_	1017.7 **±** 8.4	984.0	1012.5	1017.3	1023.0	1043.9
**Augsburg**						
DiffPressure_0_	-0.22 **±** 9.96	-31.1	-7.6	-3.9	7.8	27.1
MeanPressure_0_	1017.6 **±** 9.1	984.5	1012.2	1017.3	1023.4	1044.2

Description of atmospheric pressure parameters in hPa. Mean and standard deviation, minimum, maximum and quartiles for the observation period 2004–2009 for Munich and 2007–2009 for Augsburg.

Table 3 describes the atmospheric pressure parameters on all days, on days with onset of an MD episode and on days without onset of an MD episode, based on all 11371 patient-related data points. Because of temporal overlap between some of the patients, some meteorological data points contribute repeatedly to the analyses. Hence, *DiffPressure*_*0*_ and *MeanPressure*_*0*_ differ slightly from the values given in Table 2.

**Table 3:** Atmospheric pressure parameters before onset of MD episodes.

Variable	All days	Onset days	Other days	p-value
**Mean Air Pressure**				
MeanPressure_0_	1017.7 (8.92)	1017.9 (8.73)	1017.7 (8.93)	0.4852
MeanPressure_1_	1017.7 (8.91)	1017.9 (8.55)	1017.7 (8.93)	0.5712
MeanPressure_2_	1017.7 (8.90)	1017.5 (8.87)	1017.7 (8.91)	0.5949
MeanPressure_3_	1017.7 (8.90)	1017.6 (8.96)	1017.7 (8.90)	0.8783
**Change in Air Pressure**				
DiffPressure_0_	-0.26 (9.66)	-0.38 (9.76)	-0.25 (9.66)	0.7652
DiffPressure_1_	-0.25 (9.66)	0.62 (9.62)	-0.3 (9.66)	0.0258
DiffPressure_2_	-0.25 (9.68)	-0.41 (9.63)	-0.24 (9.69)	0.6873
DiffPressure_3_	-0.24 (9.70)	-0.27 (9.77)	-0.24 (9.7)	0.9357
**Change in Air Pressure—dichotomized**				
DiffPressure_0_ > 0	5336 (46.9%)	262 (45.4%)	5074 (47%)	0.4792
DiffPressure_1_ > 0	5345 (47.0%)	301 (52.2%)	5044 (46.7%)	0.0122
DiffPressure_2_ > 0	5344 (47.0%)	270 (46.8%)	5074 (47.0%)	0.9542
DiffPressure_3_ > 0	5339 (47.0%)	269 (46.6%)	5070 (47.0%)	0.9034

Description of atmospheric pressure parameters in hPa based on the whole observation period, based on days with a vertigo episode onset, and on days without a vertigo episode onset (other days). We report mean value and standard deviation for continuous and absolute and relative frequency for categorical variables. p-values (p) are derived from t-tests for continuous variables and chi-square tests for categorical variables and correspond to a test for difference in means on days with and without a vertigo episode onset. No statistical correction was made for multiple comparisons.

**Fig 1:**
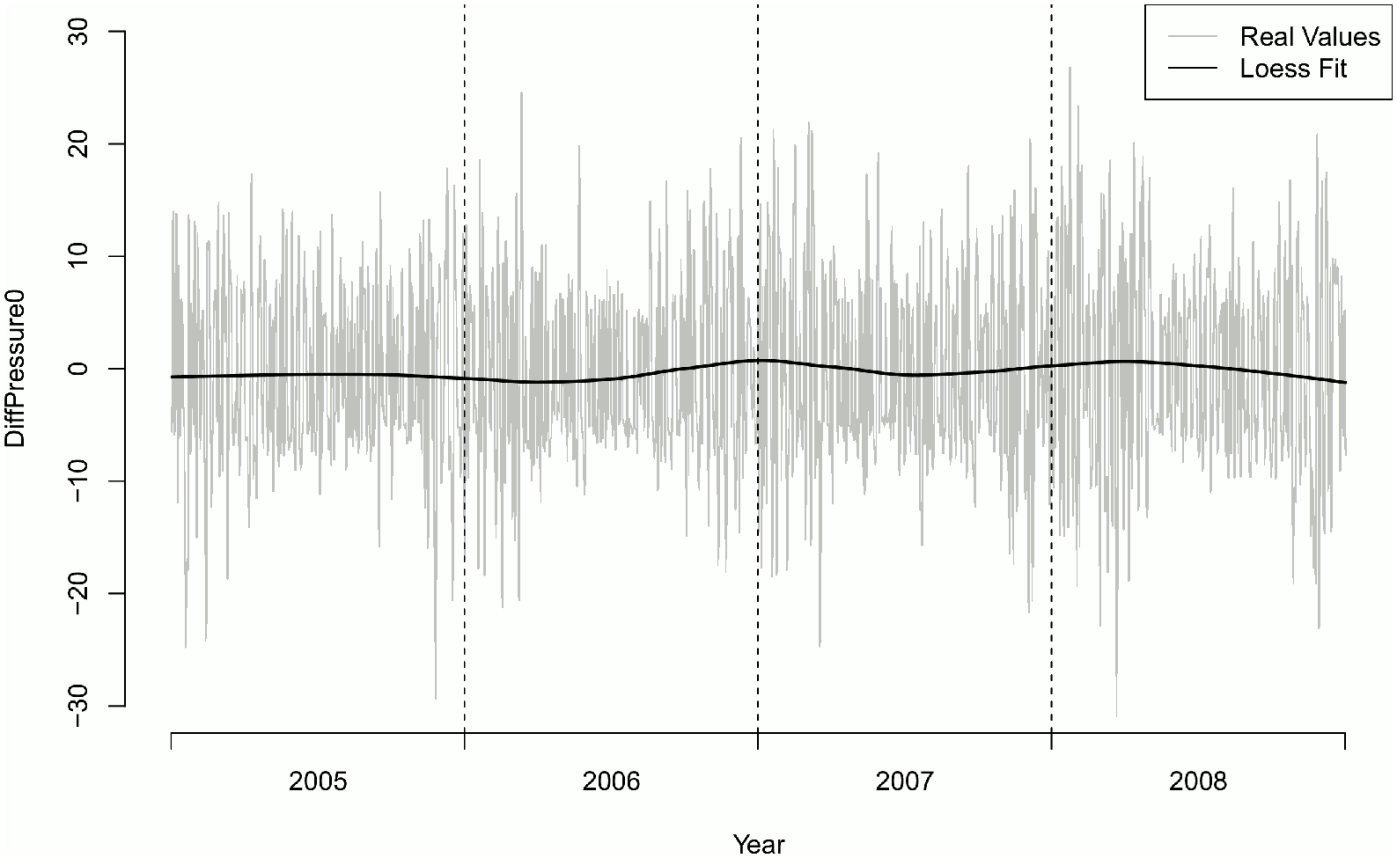
Change in air pressure over time. Station Munich-City.

**Fig 2:**
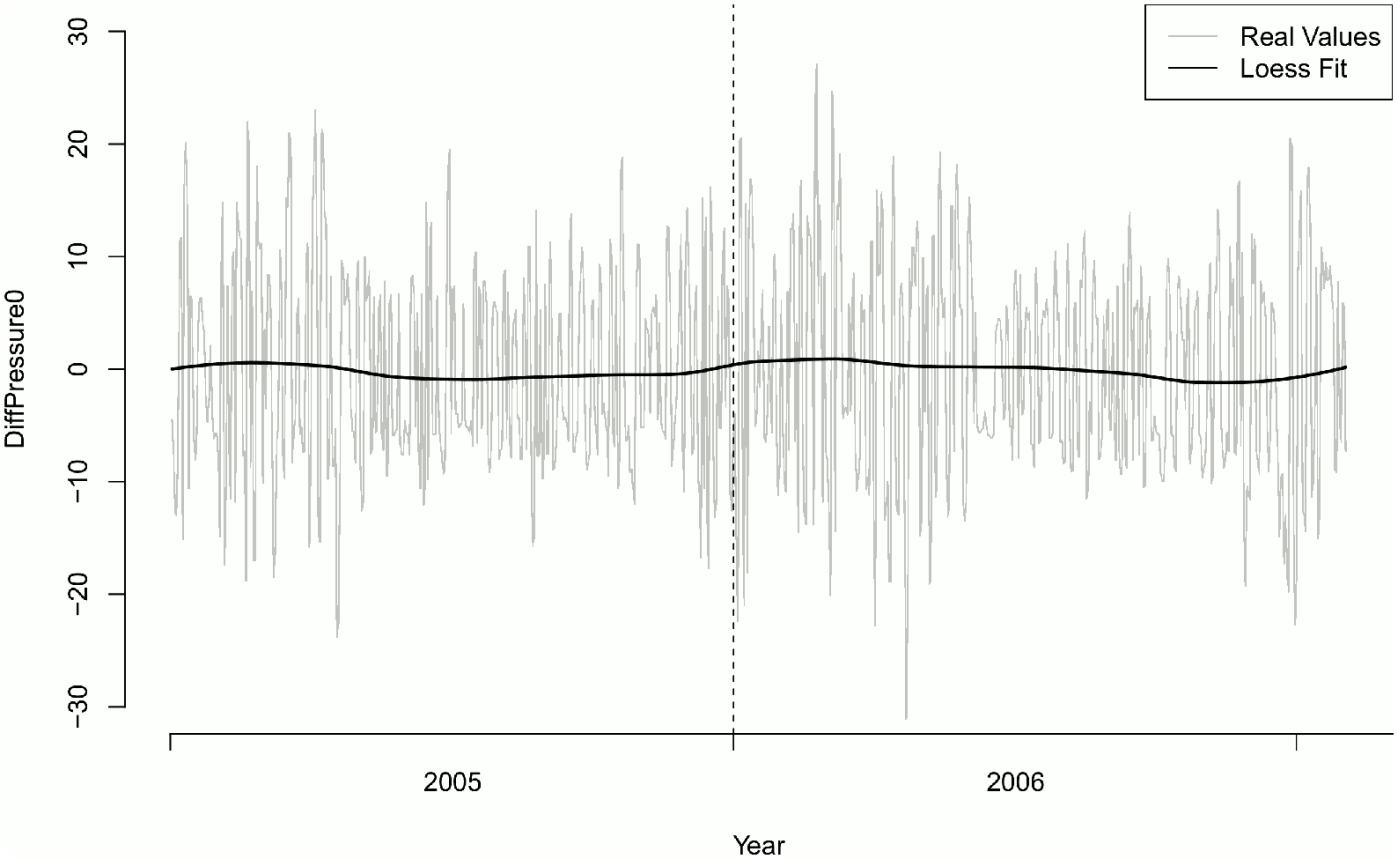
Change in air pressure over time. Station Augsburg.

**Fig 3:**
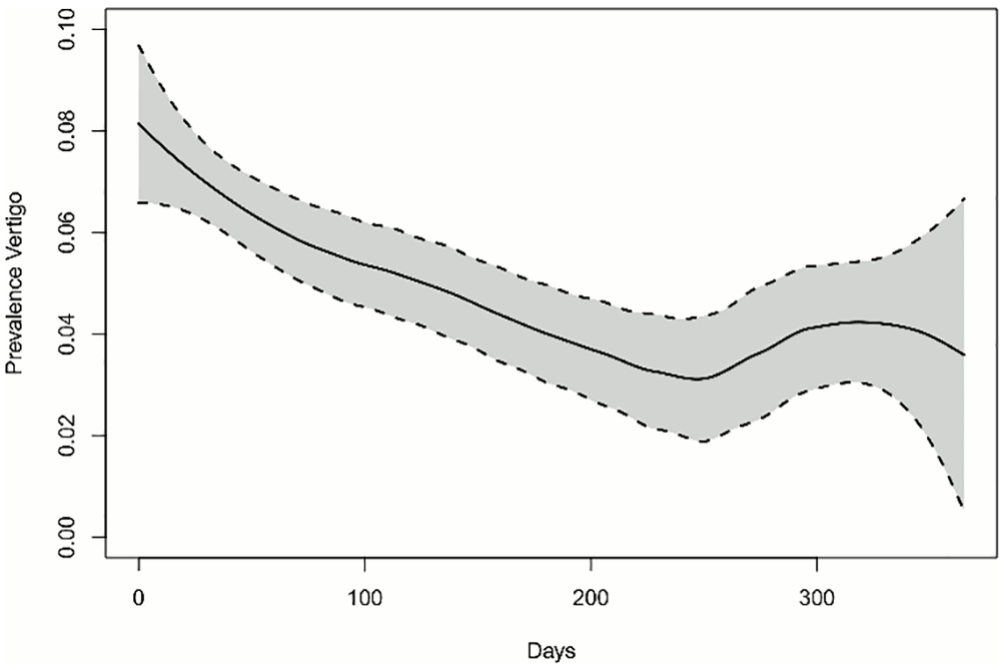
Trend of prevalence of vertigo. The prevalence of MD episodes is illustrated over the first 365 days after inclusion in the study. The black line corresponds to the loess smoother with k = 0.75. The dotted line displays the pointwise 95%-confidence intervals of the smoother. We observed a decrease in MD episode prevalence over the first 240 days and an increase thereafter.

**Fig 4:**
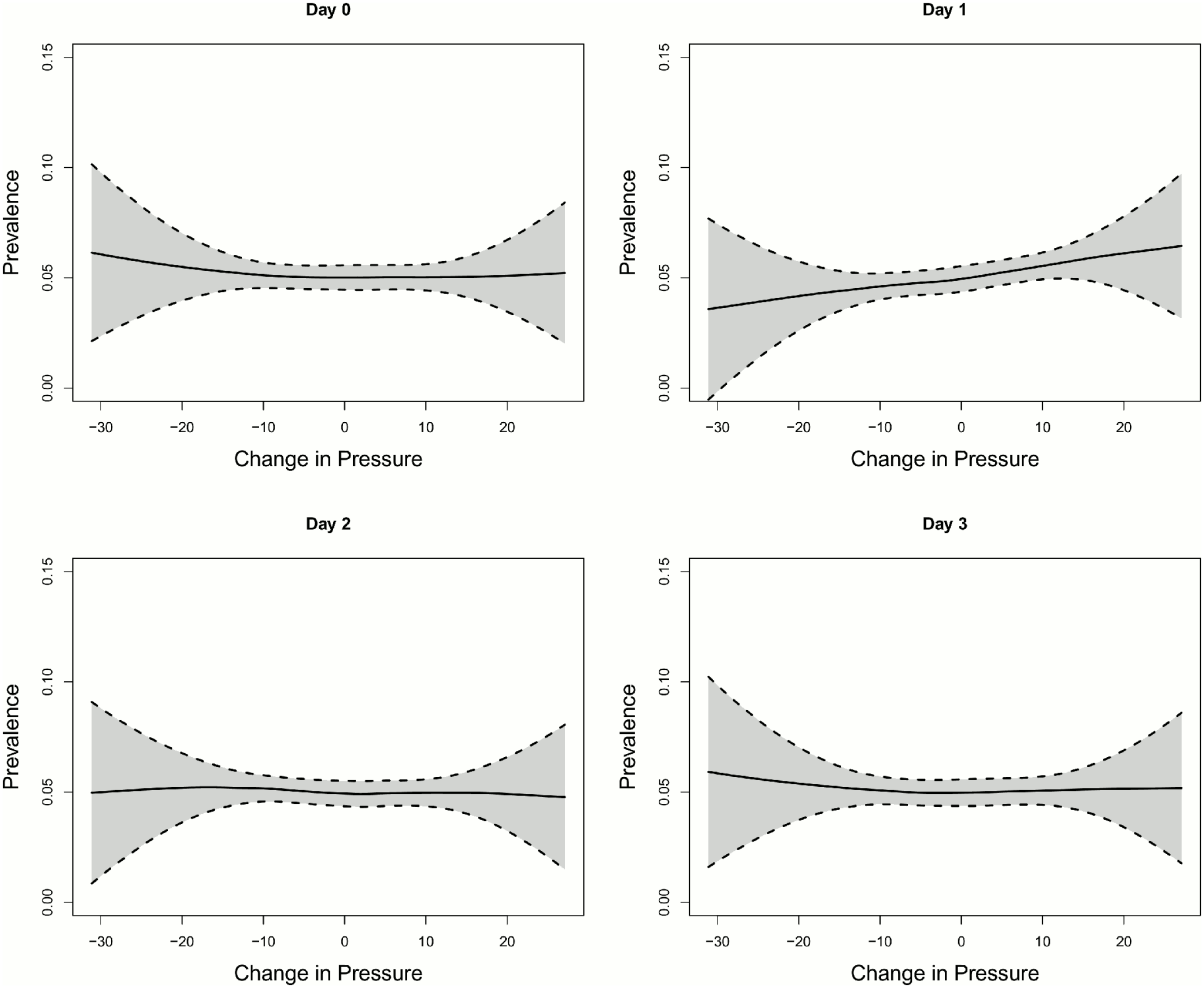
Change in atmospheric pressure versus prevalence of MD episodes. Scatter plot of change in atmospheric pressure *DiffPressure* in hPa versus prevalence of MD episodes. The black line corresponds to the loess smoother with k = 0.75. No relationship between prevalence of MD episodes and change in air pressure could be observed for air pressure values on day zero, two and three, but a roughly linear increasing relationship was found between the onset of an MD episode and change in air pressure one day before the event.

Overall number of onset of MD episodes was 577. No difference in the distribution of onsets of MD episodes could be found along the year (p = 0.4125) (Table 4). Mean change in air pressure (*DiffPressure*_*1*_) differed significantly one day prior to onset of an MD episode (p = 0.0141). There was no seasonal effect of *DiffPressure*_*0*_ (Figs 1 and 2). Frequency of MD episodes decreased during follow-up (Fig 3). Mean number of attacks per day for patients with a follow-up greater than 240 days was 0.041 compared to 0.431 (p = 0.8047). The association of *DiffPressure*_*1*_ and occurrence of an MD episode could also be verified graphically, indicating a linear relationship (Fig 4).

**Table 4:** Distribution of onsets of MD episodes along the year.

January	February	March	April	May	June	July	August	September	October	November	December
**48 (8.3%)**	**56 (9.7%)**	**55 (9.5%)**	**52 (9%)**	**47 (8.1%)**	**51 (8.8%)**	**51 (8.8%)**	**47 (8.1%)**	**31 (5.4%)**	**45 (7.8%)**	**40 (6.9%)**	**54 (9.4%)**

### Results of the generalized mixed models

The odd of the onset of an MD episode was significantly correlated with change in air pressure one day before (*DiffPressure*_*1*_, OR = 1.01). Table 5 shows the results of the generalized mixed effects models including a random participant effect. We first examined a simple unconditional model (model 1) that assumes no change over time and no difference between individuals. Adding a linear time variable yielded a superior model indicating a significant change over time (model 2). This model could be further improved by adding a quadratic time variable indicating a curvilinear change over time (model 3). Inclusion of a cubic time term did not improve the model (data not shown). Adding age and four different variables for change in air pressure (*DiffPressure*_*0*_ *–DiffPressure*_*3*_) as covariates further improved the model (OR for one year increase in age = 0.98, model 4), while adding sex as a covariate did not. Autocorrelation for air pressure parameters (*DiffPressure*_*0*_ *–DiffPressure*_*3*_) was below 0.4. Therefore, we could simultaneously include *DiffPressure*_*0*_, *DiffPressure*_*1*_, *DiffPressure*_*2*_, and *DiffPressure*_*3*_. We also included polynomials with up to 3 degrees of the pressure parameters (data not shown). This did not improve model fit, indicating a linear relationship.

**Table 5:** Results of the generalized linear mixed models presented with Odds Ratios (OR), p-values (p-val), Akaike Information Criteria (AIC) and Bayesian Information Criteria (BIC).

	*Model 1—Unconditonal*		*Model 2—Linear*		*Model 3—Quadratic*		*Model 4—Age/Gender*		*Model 5—Meteorological Model*		*Model 6—Best Model*	
	OR	p-val	OR	p-val	OR	p-val	OR	p-val	OR	p-val	OR	p-val
**Fixed Effects**												
Time in years(linear)			0.4493	< 0.0001	0.2154	< 0.0001	0.2170	< 0.0001	0.2170	< 0.0001	0.2176	< 0.0001
Time in years ^2^(quadratic)					1.8129	0.0139	1.8084	0.0145	1.8054	0.0149	1.8021	0.0151
Age in years							0.9785	0.0085	0.9795	0.0111	0.9795	0.0111
Female							0.8084	0.4193				
Diff Pressure_0_ in hPa									0.9929	0.1466		
Diff Pressure_1_ in hPa									1.0146	0.0047	1.0098	0.029
Diff Pressure_2_ in hPa									0.9917	0.1013		
Diff Pressure_3_ in hPa									1.0031	0.5249		
**Variance of Random Effect**												
Intercept	0.9281		0.8919		0.8674		0.7316		0.7338		0.7328	
**AIC**	4328.7		4302.8		4299.4		4296.7		4294.9		4292.7	
**BIC**	4343,4		4324,8		4328,7		4340,8		4361,0		4336,7	

Model 1 assumes no change over time (in years), model 2 includes a linear change in time, model 3 an additional quadratic change in time, model 4 incorporates gender and age, model 5 incorporates all atmospheric pressure changes, and model 6 represents the best model regarding AIC.

The final model contained time, age and change in air pressure one day before (Model 6). To put this into perspective, an increase of air pressure of 10hPA on Friday increased the odds for an MD episode on Saturday by 10%. An increase of 10hPa or more was observed on 269 days (15.3%) during follow-up.

As a sensitivity analysis an additional model was specified that contained a dichotomized *DiffPressure*_*1*_ variable (see Table 6). This model yielded the overall best model fit (AIC = 4291.3). The coefficient of *DiffPressure*_*1*_ > 0, corresponded to an increased risk (OR = 1.24, p-value = 0.0115). In other words, any pressure increase on Friday increased the odds for an MD episode on Saturday by 24% when compared to a pressure decrease on Friday keeping all other covariates constant.

**Table 6:** Results of the generalized linear mixed models with change in air pressure dichotomized at zero presented with Odds Ratios (OR), p-values (p-val), Akaike Information Criteria (AIC) and Bayesian Information Criteria (BIC).

	*Model 1—Meteorological Model*		*Model 2—Best Model*	
	OR	p-val	OR	p-val
**Fixed Effects**				
Time in years(linear)	0.2164	< 0.0001	0.2174	< 0.0001
Time in years ^2^(quadratic)	1.8075	0.0148	1.8037	0.0150
Age in years	0.9795	0.0112	0.9795	0.0115
Female				
Diff Pressure_0_ > 0	0.8618	0.1012		
Diff Pressure_1_ > 0	1.3138	0.0033	1.2395	0.0128
Diff Pressure_2_ > 0	0.8993	0.2540		
Diff Pressure_3_ > 0	1.0200	0.8271		
**Variance of Random Effect**				
Intercept	0.7336		0.7346	
**AIC**	4294.0		4291.3	
**BIC**	4360.0		4335.4	

Model 1 incorporates all dichotomized atmospheric pressure parameters, and model 2 represents the best model regarding AIC.

Variables for mean air pressure, weekday, season or months of study inclusion were not significant in any of the models (data not shown).

There was no association between onset of MD episodes and other weather parameters (temperature and dew point temperature). The absolute magnitude of atmospheric pressure was not correlated to the onset of an MD episode (data not shown).

Overall, the patients experienced 5351 pressure increases of which 305 were followed by an onset of a MD episode (5.7%) as opposed to 6015 pressure decreases with 272 onsets (4.5%). This corresponds to a sensitivity of 0.53 and a specificity of 0.53. The summary statistics describing the performance of DiffPressure1 > 0 as an early warning system of risk of onset of an MD episode on the individual level can be found in Table 7. The diagnostic statistics for each of the patients are displayed in Table D in S1 Appendix.

**Table 7:** Summary statistics for the quality of air pressure change as an early warning system for each of the 56 patients.

Diagnostic Parameter	Mean	Min	1st Quartile	3rd Quartile	Max
Sensitivity	54%	0%	43%	67%	100%
Specificity	54%	38%	50%	56%	77%
Positive Predictive Value	9%	0%	3%	12%	40%
Negative Predictive Value	94%	86%	91%	98%	100%

No significant association of *DiffPressure* could be found for different cut-offs for defining attack days. The detailed results of this sensitivity analysis can be seen in tables A-C in S1 Appendix.

## Discussion

To our knowledge this is the first study to show a significant association between atmospheric pressure changes and the probability of the onset of an episode of Menière’s Disease. Any ambient pressure increase raised the probability for an episode during the following day. This result was independent of other meteorological parameters such as temperature and dew point.

The existing evidence on the impact of atmospheric changes on MD is limited and variable:

While Celestino et al [24] reported that the onset of MD seemed more often in summer in Italy, Wladislavosky-Waserman et al [25] observed “a slight increase at the end of winter/beginning of spring” for the onset of MD in the North America. Mygind [26] briefly observed that “winter is the time of danger” in Sweden. Observations in Japan have noticed an increased incidence of onset of Menière’s disease after the passing of a cold front, which is in accordance with an increase in atmospheric pressure [27].

Atmospheric pressure changes are often accompanied by a more general and complex change in the weather condition. It is known that weather changes can have an impact on various aspects of health and disease. However, among the analyzed parameters, only a change in atmospheric pressure had a significant predictive value on the occurrence of an MD episode in our study.

In line with literature showing that pressure transfer from the external ear canal to the perilymph is much more effective for positive pressure changes than for negative pressure changes [17], we could confirm an increase in MD episodes after increase of atmospheric pressure but not after decrease.

In our study population living in the Metropolitan Area of Munich, 11 of the patients were living closer to the Meteorological Station of Augsburg than to Munich-City, therefore we used the Ausgsburg weather data for these patients. If the Munich weather data are used for all patients in the analysis, the observed significant effect is still present (data not shown).

The case for effects of atmospheric pressure changes on medical conditions is still not clear.

For migraine, there is no clear evidence from the literature that proves a correlation. A retrospective study in Berlin [28] on 20 migraineurs found significant effects of atmospheric pressure, temperature and humidity on occurrence of attacks in individual patients. However, for the overall population, pressure changes did not have a significant effect. A prospective study in Vienna [29] on 238 migraineurs analyzed the correlation between attacks and 11 different meteorological parameters as well as 17 synoptic weather conditions and, after correction for multiple testing, did not find any statistically significant correlation.

Several studies showed an influence of atmospheric pressure changes on subarachnoid haemorrhage [30–33]. For acute glaucoma, among a great variety of meteorological parameters, a significant correlation was only found with atmospheric pressure [34].

An analysis of the occurrence of another inner ear disorder, idiopathic sudden sensorineural hearing loss, however, could not find a correlation with either seasonal rhythms, temperature, pressure, temperature changes or pressure changes [35].

Pereira et al [36] examined the seasonality of the incidence of cases presenting to a large emergency department with dizziness and vertigo in the tropical region of southern Brazil. For the symptom “vertigo” (which would rather correspond to our definition of MD attacks than “dizziness”), they found a significant seasonality pattern, with a peak in late winter. According to their focus on seasonality, climatic parameters like temperature, atmospheric pressure, sunshine, humidity and rainfall were averaged monthly and further analyzed for correlations with the incidence of vertigo. No correlation between vertigo and atmospheric pressure was found, but a negative correlation with rainfall and humidity. These findings seem to contrast with the results in the present study. It has to be kept in mind, however, that besides the differences in treatment of the meteorological variables (mean pressure vs pressure changes), their study examined exclusively emergency department visits due to vertigo symptoms which 1) are not limited to MD patients but include all etiologies of vertigo and 2) would probably only include the initial or most severe manifestations of MD, since most MD patients would not visit the emergency department every time when suffering an MD attack.

Effects of weather on human pathologies are often studied using a complex meteorological classification of atmospheric conditions. Our approach differed insofar as we hypothesized a specific pathophysiologic pathway by postulating that changes in ambient pressure are transmitted to the inner ear fluid system.

It is interesting to note that the effect of atmospheric pressure changes identified in this study occurred on the day before the onset of the MD episode and not on the same day. Keeping in mind that the subjects recorded only the day of the MD episodes and not the precise time at which the episode started, it is not possible to determine the exact time delay between the exposure to a pressure change and the attack. Nevertheless, this observed lag of about one day points towards an interval of at least several hours. Our knowledge on the response of the inner ear to pressure changes is limited. Experimental evidence suggests that 1) rapid pressure increases in the external ear canal above 20 hPa within tens of seconds are transmitted to the inner ear perilymph, but also compensated in a similar time frame [37] and that 2) so-called atmospheric pressure fluctuations with minute amplitudes of a few tens of pascals and very low frequencies in the far infrasound range of milliHertz are readily transmitted to the middle ear via the pars flaccida of the eardrum. However, the effect of pressure changes occurring over a longer time span, i.e. hours, on inner ear homoeostasis has not yet been investigated physiologically, to our knowledge. It is conceivable, nevertheless, that such a slow disturbance calls for a slow compensatory mechanism. Therefore, the observed lag in our study may be interpreted as a hint towards the involvement of paracrine factors like the Atrial Natriuretic Peptide (ANP) system, as opposed to probably faster-acting hydromechanical factors in connection with Bast’s valve. Supporting this thesis, a recent study in mice has shown the inhibitory effect of ANP on epithelial Sodium channel (ENaC) expression in the inner ear, with a maximum after 2 hours [38]. Inhibition of ENaC would be expected to lead to an increase in endolymphatic Na+ concentration, subsequently resulting in an elevation in endolymph volume and therefore aggravation of endolymphatic hydrops.

The observed trend of amelioration of vertigo attacks over time—after the first visit to the tertiary referral centre—is probably at least partially due to a placebo effect. Very recently, a large prospective randomized placebo-controlled trial that compared Betahistine with placebo treatment has demonstrated that both treatment groups improved similarly during the 12-month observation period [39]. Therefore, our study population can be regarded in retrospect as having been treated with a placebo.

### Limitations

Our data do not allow to exclude the possibility that the pressure increase causes a physiological response, but at a site distant from the inner ear which in turn may trigger an MD attack by another mechanism, e.g. involving circulatory or hormonal perturbations. However, even though the physiological pathways leading from change in air pressure to the onset of an MD episode may not be fully understood, the effect itself cannot be denied and air pressure may still be regarded as an early warning system.

While it might be seen as a limitation that we used over all meteorological parameters and not individual exposure as predictors of risk, this approach excluded information and expectation bias. The exact location of an individual subject at the time of vertigo attacks may have an influence on the occurrence of attacks. This information was not routinely recorded in the symptom diaries. This is mostly due to our intention to keep the patient self-reported diaries as simple as possible, since in our experience the compliance of patients to fill in daily symptom reports and their reliability declines rapidly as the complexity of the symptom diary increases.

Our study examined patients from one geographic region with specific weather conditions. In the future, these results should ideally be reproduced in other geographic regions as well.

We based our analyses on patients referred to a tertiary care vertigo and balance clinic. While our patient population consists of all 4 disease stages, this sample might still contain a bias towards more severe cases of MD than an average MD population.

As we did not record the day time of the beginning of an MD episode, we could not precisely estimate the time-shifted impact of meteorological parameters.

## Conclusion

Our study found a significant correlation between an increase in atmospheric pressure and the probability of an MD episode onset on the next day. On the basis of this finding, it is conceivable to develop a warning system for MD patients which could help them in planning their activities for the next day, coping with this incurable disease and reducing their fear of the next unpredictable vertigo episode. Furthermore, our findings support the notion that MD patients suffer from a reduced ability of their inner ear to maintain pressure homeostasis when challenged with atmospheric pressure increases. Therefore future research efforts to elucidate the mechanisms of inner ear pressure homeostasis are warranted in order to ultimately decipher the pathophysiology of MD and identify targets for therapeutic interventions.

## Supporting Information

S1 Appendix **Table A** Results of the generalized linear mixed models—attack day was defined as a day with an intensity of one or higher.**Table B** Results of the generalized linear mixed models—attack day was defined as a day with an intensity of three or higher.**Table C** Results of the generalized linear mixed models—attack day was defined as a day with an intensity of four or higher.**Table D** Individual diagnostic statistics (Sensitivity (SENS), Specificity (SPEC), Positive Predictive Value (PPV), Negative Predictive Value (NPV)) for the quality of air pressure change as an early warning system for each of the 56 patients together with the number of MD episodes.
(DOCX)

S1 Fig Autocorrelation of different orders of mean air pressure (left) and change in air pressure (right). The y-axis shows the correlation coefficient of different orders (Lag) up to a 30 days.
(TIFF)

S1 Dataset atmospheric pressure values for meteorological station Augsburg. (XLSX)

S2 Dataset atmospheric pressure values for meteorological station Munich-City. (XLSX)

S3 Dataset Closest meteorological station for each patient. (XLSX)

S4 Dataset Daily vertigo scores of all patients. (XLSX)
