# Supplementary material for: Atmospheric Pressure and Onset of Episodes of Menière’s Disease - A Repeated Measures Study
Source: PLoS One. 2016 Apr 20;11(4):e0152714. doi: 10.1371/journal.pone.0152714 (PMC4838262; doi:10.1371/journal.pone.0152714)
Supplement: S1 Appendix — Table A Results of the generalized linear mixed models—attack day was defined as a day with an intensity of one or higher.Table B Results of the generalized linear mixed models—attack day was defined as a day with an intensity of three or higher.Table C Results of the generalized linear mixed models—attack day was defined as a day with an intensity of four or higher.Table D Individual diagnostic statistics (Sensitivity (SENS), Specificity (SPEC), Positive Predictive Value (PPV), Negative Predictive Value (NPV)) for the quality of air pressure change as an early warning system for each of the 56 patients together with the number of MD episodes. (DOCX) [file pone.0152714.s001.docx]

**Electronic Appendix**

**Atmospheric pressure and onset of episodes of Menière’s Disease - a repeated measures study**

**Table A**  Results of the generalized linear mixed models – attack day was defined as a day with an intensity of one or higher.

|  | ***Model 1***  ***Unconditonal*** | | ***Model 2***  ***Linear*** | | ***Model 3***  ***Quadratic*** | | ***Model 4***  ***Age/Gender*** | | ***Model 5***  ***Meteorological Model*** | | ***Model 6***  ***Best Model*** | |
| --- | --- | --- | --- | --- | --- | --- | --- | --- | --- | --- | --- | --- |
|  | **OR** | **p-val** | **OR** | **p-val** | **OR** | **p-val** | **OR** | **p-val** | **OR** | **p-val** | **OR** | **p-val** |
| **Fixed Effects** |  |  |  |  |  |  |  |  |  |  |  |  |
| Time in years (linear) |  |  | 0.5323 | < 0.0001 | 0.4483 | 0.0330 | 0.4594 | 0.0387 | 0.4601 | 0.0391 | 0.4600 | 0.0390 |
| Time in years ^2^(quadratic) |  |  |  |  | 1.1502 | 0.6207 | 1.1352 | 0.6536 | 1.1332 | 0.6583 | 1.1336 | 0.6574 |
| Age in years |  |  |  |  |  |  | 0.9825 | 0.0331 | 0.9827 | 0.0351 | 0.9827 | 0.0341 |
| Female |  |  |  |  |  |  | 0.9407 | 0.8050 |  |  |  |  |
| Diff Pressure_0_ in hPa |  |  |  |  |  |  |  |  | 0.9963 | 0.4477 |  |  |
| Diff Pressure_1_ in hPa |  |  |  |  |  |  |  |  | 1.0052 | 0.3069 | 1.0024 | 0.5919 |
| Diff Pressure_2_ in hPa |  |  |  |  |  |  |  |  | 0.9929 | 0.1646 |  |  |
| Diff Pressure_3_ in hPa |  |  |  |  |  |  |  |  | 0.9996 | 0.9352 |  |  |
| **Variance of Random Effect** | | | | | | | | |  |  |  |  |
| Intercept | 0.6137 |  | 0.6482 |  | 0.6468 |  | 0.5485 |  | 0.5508 |  | 0.5493 |  |
| **AIC** | 4182.5 |  | 4165.9 |  | 4167.7 |  | 4167.5 |  | 4170.9 |  | 4167.3 |  |
| **BIC** | 4196.5 |  | 4186.0 |  | 4195.8 |  | 4209.6 |  | 4234.1 |  | 4209.4 |  |

**Table B**  Results of the generalized linear mixed models – attack day was defined as a day with an intensity of three or higher.

|  | ***Model 1***  ***Unconditonal*** | | ***Model 2***  ***Linear*** | | ***Model 3***  ***Quadratic*** | | ***Model 4***  ***Age/Gender*** | | ***Model 5***  ***Meteorological Model*** | | ***Model 6***  ***Best Model*** | |
| --- | --- | --- | --- | --- | --- | --- | --- | --- | --- | --- | --- | --- |
|  | **OR** | **p-val** | **OR** | **p-val** | **OR** | **p-val** | **OR** | **p-val** | **OR** | **p-val** | **OR** | **p-val** |
| **Fixed Effects** |  |  |  |  |  |  |  |  |  |  |  |  |
| Time in years (linear) |  |  | 0.5036 | < 0.0001 | 0.2103 | < 0.0001 | 0.2153 | < 0.0001 | 0.2156 | < 0.0001 | 0.2161 | < 0.0001 |
| Time in years ^2^(quadratic) |  |  |  |  | 1.8959 | 0.0056 | 1.8791 | 0.0064 | 1.8736 | 0.0067 | 1.8688 | 0.0068 |
| Age in years |  |  |  |  |  |  | 0.9741 | 0.0061 | 0.9748 | 0.0069 | 0.9749 | 0.0070 |
| Female |  |  |  |  |  |  | 0.8556 | 0.6110 |  |  |  |  |
| Diff Pressure_0_ in hPa |  |  |  |  |  |  |  |  | 0.9978 | 0.7089 |  |  |
| Diff Pressure_1_ in hPa |  |  |  |  |  |  |  |  | 1.0114 | 0.0591 | 1.0093 | 0.0775 |
| Diff Pressure_2_ in hPa |  |  |  |  |  |  |  |  | 0.9927 | 0.2195 |  |  |
| Diff Pressure_3_ in hPa |  |  |  |  |  |  |  |  | 0.9983 | 0.7641 |  |  |
| **Variance of Random Effect** | | | | | | | | |  |  |  |  |
| Intercept | 1.1761 |  | 1.1883 |  | 1.1628 |  | 0.9795 |  | 0.9793 |  | 0.9769 |  |
| **AIC** | 3340.1 |  | 3325.2 |  | 3320.4 |  | 3317.4 |  | 3318.7 |  | 3314.6 |  |
| **BIC** | 3355.0 |  | 3347.6 |  | 3350.3 |  | 3362.2 |  | 3385.9 |  | 3359.5 |  |

**Table C** Results of the generalized linear mixed models – attack day was defined as a day with an intensity of four or higher.

|  | ***Model 1***  ***Unconditonal*** | | ***Model 2***  ***Linear*** | | ***Model 3***  ***Quadratic*** | | ***Model 4***  ***Age/Gender*** | | ***Model 5***  ***Meteorological Model*** | | ***Model 6***  ***Best Model*** | |
| --- | --- | --- | --- | --- | --- | --- | --- | --- | --- | --- | --- | --- |
|  | **OR** | **p-val** | **OR** | **p-val** | **OR** | **p-val** | **OR** | **p-val** | **OR** | **p-val** | **OR** | **p-val** |
| **Fixed Effects** |  |  |  |  |  |  |  |  |  |  |  |  |
| Time in years (linear) |  |  | 0.9301 | 0.814 | 1.3825 | 0.7038 | 1.4588 | 0.6583 | 1.4791 | 0.6482 | 1.4628 | 0.6561 |
| Time in years ^2^(quadratic) |  |  |  |  | 0.7529 | 0.6193 | 0.7286 | 0.5803 | 0.7199 | 0.5691 | 0.7282 | 0.5796 |
| Age in years |  |  |  |  |  |  | 0.9665 | 0.0666 | 0.9666 | 0.0579 | 0.966 | 0.0576 |
| Female |  |  |  |  |  |  | 1.0754 | 0.9033 |  |  |  |  |
| Diff Pressure_0_ in hPa |  |  |  |  |  |  |  |  | 0.9844 | 0.1724 |  |  |
| Diff Pressure_1_ in hPa |  |  |  |  |  |  |  |  | 0.9939 | 0.6299 | 1.0034 | 0.7487 |
| Diff Pressure_2_ in hPa |  |  |  |  |  |  |  |  | 1.0210 | 0.0861 |  |  |
| Diff Pressure_3_ in hPa |  |  |  |  |  |  |  |  | 0.9671 | 0.0036 |  |  |
| **Variance of Random Effect** | | | | | | | | |  |  |  |  |
| Intercept | 2.7084 |  | 2.7059 |  | 2.7192 |  | 2.4769 |  | 2.3843 |  | 2.4878 |  |
| **AIC** | 1003.7 |  | 1005.7 |  | 1007.4 |  | 1007.7 |  | 1002.2 |  | 1007.7 |  |
| **BIC** | 1018.8 |  | 1028.3 |  | 1037.6 |  | 1053.0 |  | 1070.2 |  | 1052.9 |  |

**Table D**  Individual diagnostic statistics (Sensitivity (SENS), Specificity (SPEC), Positive Predictive Value (PPV), Negative Predictive Value (NPV)) for the quality of air pressure change as an early warning system for each of the 56 patients together with the number of MD episodes.

| **Patient** | **SENS** | **SPEC** | **PPV** | **NPV** | **MD Episodes** |
| --- | --- | --- | --- | --- | --- |
| 1 | 66.7% | 52.0% | 7.8% | 96.2% | 9 |
| 2 | 52.6% | 56.0% | 9.5% | 93.1% | 19 |
| 3 | 72.7% | 57.9% | 8.3% | 97.6% | 11 |
| 4 | 66.7% | 76.9% | 40.0% | 90.9% | 3 |
| 5 | 22.2% | 56.5% | 1.5% | 96.0% | 9 |
| 6 | 66.7% | 56.8% | 2.1% | 99.2% | 6 |
| 7 | 66.7% | 55.4% | 2.6% | 98.9% | 3 |
| 8 | 57.1% | 56.8% | 4.1% | 97.6% | 14 |
| 9 | 52.2% | 57.5% | 13.2% | 90.7% | 23 |
| 10 | 25.0% | 61.2% | 1.5% | 97.2% | 4 |
| 11 | 42.9% | 56.3% | 3.8% | 96.1% | 14 |
| 12 | 37.5% | 62.5% | 11.8% | 88.2% | 16 |
| 13 | 42.9% | 57.0% | 12.3% | 87.6% | 21 |
| 14 | 33.3% | 55.7% | 6.1% | 90.7% | 15 |
| 15 | 50.0% | 58.3% | 4.8% | 96.6% | 4 |
| 16 | 50.0% | 56.1% | 1.0% | 99.2% | 4 |
| 17 | 75.0% | 54.7% | 4.6% | 98.7% | 8 |
| 18 | 33.3% | 52.4% | 1.0% | 98.2% | 3 |
| 19 | 50.0% | 55.5% | 11.5% | 90.6% | 20 |
| 20 | 0% | 58.3% | 0% | 98.9% | 1 |
| 21 | 45.0% | 50.8% | 4.6% | 94.6% | 20 |
| 22 | 80.0% | 55.6% | 4.4% | 99.1% | 5 |
| 23 | 66.7% | 53.1% | 9.3% | 95.7% | 15 |
| 24 | 55.0% | 51.2% | 13.2% | 89.4% | 60 |
| 25 | 100% | 49.6% | 0.8% | 100% | 1 |
| 26 | 48.4% | 49.6% | 7.8% | 91.6% | 31 |
| 27 | 66.7% | 44.9% | 11.6% | 92.5% | 15 |
| 28 | 47.2% | 49.6% | 12.1% | 86.5% | 36 |
| 29 | - | 49.5% | - | - | 0 |
| 30 | 50.0% | 48.5% | 13.6% | 85.7% | 16 |
| 31 | 55.6% | 50.0% | 2.9% | 97.6% | 9 |
| 32 | - | 50.0% | - | - | 0 |
| 33 | 66.7% | 75.0% | 33.3% | 92.3% | 3 |
| 34 | 50.0% | 50.2% | 0.6% | 99.4% | 2 |
| 35 | 50.0% | 44.1% | 10.8% | 86.7% | 8 |
| 36 | 57.1% | 51.0% | 5.3% | 96.2% | 7 |
| 37 | 50.0% | 42.0% | 9.4% | 87.5% | 6 |
| 38 | 61.1% | 53.8% | 8.2% | 95.3% | 18 |
| 39 | 100% | 51.6% | 2.7% | 100% | 3 |
| 40 | 64.3% | 51.1% | 6.1% | 96.6% | 14 |
| 41 | 28.6% | 53.8% | 3.1% | 93.7% | 14 |
| 42 | - | 50.7% | - | - | 0 |
| 43 | 50.0% | 50.5% | 5.3% | 94.8% | 12 |
| 44 | - | 49.7% | - | - | 0 |
| 45 | 53.8% | 53.1% | 13.2% | 89.7% | 13 |
| 46 | 100% | 55.9% | 11.8% | 100% | 2 |
| 47 | 0% | 50.0% | 0% | 97.3% | 2 |
| 48 | 42.9% | 50.5% | 5.2% | 93.3% | 14 |
| 49 | 37.5% | 45.5% | 7.7% | 85.7% | 8 |
| 50 | 75.0% | 50.0% | 16.7% | 93.8% | 4 |
| 51 | 0% | 52.3% | 0% | 95.7% | 2 |
| 52 | 100% | 37.5% | 16.7% | 100% | 1 |
| 53 | 66.7% | 5.09% | 7.4% | 97.3% | 3 |
| 54 | 70.0% | 53.7% | 6.5% | 97.5% | 10 |
| 55 | 42.9% | 55.3% | 12.5% | 86.7% | 7 |
| 56 | 66.7% | 63.4% | 28.6% | 89.7% | 9 |
